# Neurological Manifestations of Hemolytic Uremic Syndrome: A Comprehensive Review

**DOI:** 10.3390/brainsci15070717

**Published:** 2025-07-04

**Authors:** Una Tonkovic, Marko Bogicevic, Aarish Manzar, Nikola Andrejic, Aleksandar Sic, Marko Atanaskovic, Selena Gajić, Ana Bontić, Sara Helena Ksiazek, Ana Mijušković, Nikola M. Stojanović, Marko Baralić

**Affiliations:** 1 Faculty of Medicine, University of Belgrade, 11000 Belgrade, Serbia; unaunattt@gmail.com (U.T.); anikola99@yahoo.com (N.A.);; 2 Chicago College of Osteopathic Medicine, Midwestern University, Downers Grove, IL 60515, USA; marko.bogicevic@midwestern.edu; 3 Department of Internal Medicine, Ziauddin Medical College, Karachi 75500, Pakistan; 4 Neurology Clinic, University Clinical Centre of Serbia, Dr Subotica Starijeg Str. 6, 11000 Belgrade, Serbia; 5 Faculty of Medicine, University of Novi Sad, 21000 Novi Sad, Serbia; atanaskovic.marko5@gmail.com; 6 Izola General Hospital, 6310 Izola, Slovenia; 7 Clinic of Nephrology, University Clinical Centre of Serbia, Pasterova Str. 2, 11000 Belgrade, Serbia; 8 6th Medical Department of Internal Medicine with Nephrology & Dialysis, Clinic Ottakring, 1160 Vienna, Austria; 9 Centre for Mental Health Protection, University Clinical Centre Niš, Bulevar Dr. Zorana Đinđića Str. 48, 18000 Niš, Serbia

**Keywords:** hemolytic uremic syndrome, atypical HUS, STEC-HUS, pediatric neurology, nephrology, thrombotic microangiopathy, neuroimaging, biomarkers

## Abstract

Hemolytic uremic syndrome (HUS), a thrombotic microangiopathy primarily affecting the kidneys, can also involve the central nervous system (CNS), often leading to significant morbidity and mortality. Neurologic manifestations are among the most severe extra-renal complications, particularly in children and during outbreaks of Shiga toxin-producing Escherichia coli (STEC)-associated HUS (typical (tHUS)). This review explores the clinical spectrum, pathophysiology, diagnostic workup, and age-specific outcomes of neurologic involvement in both typical (tHUS) and atypical (aHUS). Neurologic complications occur in up to 11% of pediatric and over 40% of adult STEC-HUS cases in outbreak settings. Presentations include seizures, encephalopathy, focal deficits, movement disorders, and posterior reversible encephalopathy syndrome (PRES). Magnetic resonance imaging (MRI) commonly reveals basal ganglia or parieto-occipital lesions, though subtle or delayed findings may occur. Laboratory workup typically confirms microangiopathic hemolytic anemia (MAHA), thrombocytopenia, and kidney damage, with additional markers of inflammation or metabolic dysregulation. Eculizumab is the first-line treatment for aHUS with CNS involvement, while its utility in STEC-HUS remains uncertain. Although many children recover fully, those with early CNS involvement are at greater risk of developing epilepsy, cognitive delays, or fine motor deficits. Adults may experience lingering neurocognitive symptoms despite apparent clinical recovery. Differences in presentation and imaging findings between age groups emphasize the need for tailored diagnostic and therapeutic strategies. Comprehensive neurorehabilitation and long-term follow-up are crucial for identifying residual deficits. Continued research into predictive biomarkers, neuroprotective interventions, and standardized treatment protocols is needed for improving outcomes in HUS patients with neurological complications.

## 1. Introduction

Neurological injury represents one of the most severe yet frequently underrecognized complications of hemolytic uremic syndrome (HUS) in pediatric nephrology but also the adult population. HUS is a form of thrombotic microangiopathy (TMA), characterized by the clinical triad of microangiopathic hemolytic anemia (MAHA), thrombocytopenia, and acute kidney injury (AKI). It is classically categorized into two main types: typical HUS (tHUS), most commonly associated with Shiga toxin-producing *Escherichia coli* (STEC-HUS), and atypical HUS (aHUS), which results from dysregulation of the alternative complement pathway. While both forms lead to endothelial injury and microvascular thrombosis, their etiologies differ markedly. STEC-HUS is toxin-mediated, whereas aHUS is driven by uncontrolled complement activation, resulting in distinct implications for neurological involvement [1].

Typical HUS, also known as diarrhea-associated HUS (D+ HUS), accounts for approximately 90% of pediatric cases. In contrast, atypical HUS can occur at any age and is commonly linked to genetic mutations or acquired abnormalities in complement regulation [2]. Epidemiologically, HUS predominantly affects children under the age of five. However, notable exceptions exist, such as the 2011 European outbreak of *E. coli* O104:H4, which primarily affected adults and was associated with unusually high rates of systemic and neurological complications [3].

Although not present in all patients, neurological manifestations in HUS are clinically significant due to their contribution to both morbidity and mortality. Historically, central nervous system (CNS) involvement has been documented in up to 50% of STEC-HUS cases, representing the leading cause of death during the acute phase of illness [4]. Neurological symptoms vary widely, ranging from seizures and encephalopathy to cortical blindness and stroke-like syndromes. While advances in intensive care and targeted therapies have reduced acute mortality, timely recognition and management of neurological complications remain essential. A recent pediatric cohort study found neurological involvement in 11% of HUS cases, with most patients achieving favorable outcomes following early intervention [5].

This review aims to provide a structured and comprehensive overview of the pathophysiology and clinical spectrum of neurological complications in HUS, with a particular focus on distinguishing typical and atypical forms. Additionally, it examines emerging diagnostic tools, therapeutic strategies, and long-term prognostic considerations, highlighting areas for future research aimed at optimizing neurological outcomes.

## 2. Pathophysiology of HUS and Neurological Involvement

### 2.1. Shiga Toxin-Mediated Endothelial Injury and Microangiopathy

Endothelial injury induced by Shiga toxin represents the central pathological event in Shiga toxin-producing *Escherichia coli* STEC-HUS. This process initiates a cascade culminating in systemic TMA. Shiga toxin (Stx), produced by enterohemorrhagic *E. coli* (EHEC), enters the systemic circulation following gastrointestinal infection [6]. Although the precise mechanism of intestinal translocation remains incompletely understood, it is believed that the B subunit of the toxin binds to Gb3/CD77 receptors on intestinal epithelial or Paneth cells, facilitating absorption into the bloodstream [7]. Once in circulation, Shiga toxin binds to neutrophils (Ne), platelets (Plt), erythrocytes (Er), and monocytes (Mo), triggering the release of microvesicles, small membrane-bound particles that serve as transporters of the toxin to organs expressing high levels of Gb3 receptors, especially the kidneys [8].

In kidneys’ microvasculature, these microvesicles are internalized by endothelial cells in glomerular and peritubular capillaries via endocytosis. Within the cells, the enzymatically active A subunit of the toxin is released, cleaving the 28S ribosomal RNA of the 60S ribosomal subunit and irreversibly halting protein synthesis. This triggers cellular apoptosis and widespread endothelial injury [9,10].

The damaged endothelium exposes subendothelial collagen and von Willebrand factor (vWF), promoting Plt adhesion and activation. Concurrently, Shiga toxin upregulates the expression of proinflammatory cytokines (TNF-α, IL-6, IL-8) and tissue factor (TF), fostering thrombin generation and fibrin deposition [11]. These events establish a prothrombotic milieu, leading to microvascular occlusion, particularly in renal vessels, and resulting in ischemia and AKI [12].

Moreover, the obstruction of microvessels generates shear stress that fragments Er-producing mechanical hemolysis. This is evident on peripheral blood smears as schistocytes and is reflected biochemically by elevated lactate dehydrogenase (LDH) levels [13]. Although kidney injury predominates, other organs, including the central nervous system, may also be affected by this systemic microangiopathic process [8]. 

While STEC-HUS is primarily mediated by bacterial toxin, aHUS results from complement dysregulation, producing similar microvascular damage through distinct immunological mechanisms.

### 2.2. Complement Activation in Atypical HUS

Atypical hemolytic uremic syndrome (aHUS) arises from dysfunction in the regulation of the alternative complement pathway, resulting in uncontrolled complement activation and subsequent TMA. Unlike STEC-HUS, aHUS is typically associated with genetic mutations in complement-regulating proteins, including complement factor H (CFH), factor I (CFI), membrane cofactor protein (CD46/MCP), complement component C3, or factor B (CFB), or with acquired autoantibodies against CFH that impair regulatory control [14,15].

Under physiological conditions, the alternative complement pathway is constitutively active at low levels. In aHUS, defective regulatory proteins fail to control this activation, allowing persistent formation of the C3 convertase complex (C3bBb). This leads to excessive generation of the proinflammatory mediators C3a and C5a and to the assembly of the membrane attack complex (MAC, C5b-9) on endothelial surfaces [16]. The deposition of C5b-9 induces endothelial activation and dysfunction, characterized by loss of thrombomodulin, increased expression of tissue factor, and release of large vWF multimers, all contributing to Plt aggregation and microthrombosis [11].

The particular vulnerability of glomerular endothelial cells remains incompletely understood. One hypothesis attributes this sensitivity to their fenestrated structure and the relative paucity of local complement regulatory proteins within the glomerular basement membrane (GBM). Although renal vasculature is the primary target, systemic complement overactivation can also affect other organs. Neurological complications such as posterior reversible encephalopathy syndrome (PRES) and seizures, cardiac involvement from microvascular thrombosis, and intestinal ischemia have all been documented in aHUS [17].

In cases of suspected pregnancy-associated aHUS, distinguishing it from conditions such as preeclampsia and HELLP syndrome is challenging due to overlapping clinical features. Biomarkers related to angiogenesis have shown diagnostic value in this setting. Low levels of placental growth factor (PlGF) and an elevated soluble fms-like tyrosine kinase-1 to PlGF (sFlt-1/PlGF) ratio are more characteristic of preeclampsia, whereas their preservation may indicate complement-mediated aHUS. Ex vivo assays detecting C5b-9 deposition on human microvascular endothelial cells (HMEC-1) are emerging as promising tools for confirming complement activation [18].

### 2.3. Mechanisms of Central Nervous System Injury in Hemolytic Uremic Syndrome

Neurological complications represent some of the most severe clinical manifestations of HUS, occurring in approximately 20–50% of cases and contributing significantly to mortality during the acute phase of illness [19]. These complications result from three principal, and often overlapping, pathological mechanisms: endothelial injury, microvascular thrombosis, and metabolic encephalopathy [20]. The relative contribution of each mechanism varies between STEC-HUS and aHUS, influencing both clinical presentation and therapeutic strategy.

#### 2.3.1. Endothelial Injury

**Figure 1:**
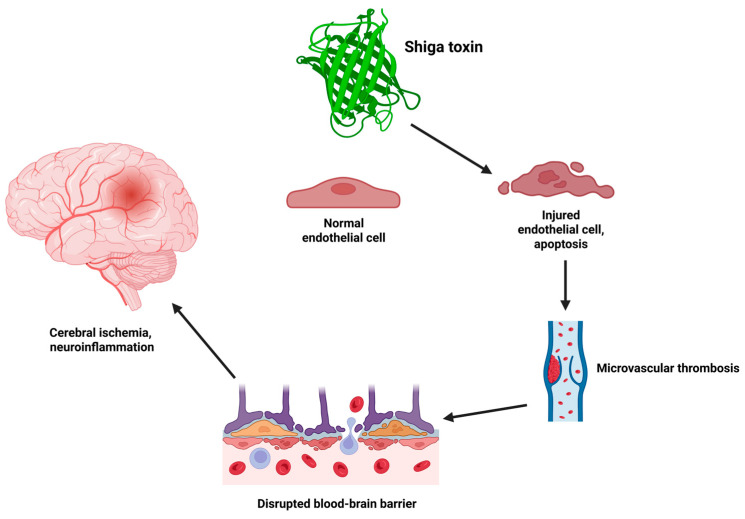
Shiga toxin-induced endothelial injury leading to cerebral microvascular thrombosis and ischemia. Created in BioRender online application, available at https://app.biorender.com/ (accessed on 10 June 2025).

Endothelial injury in HUS arises through distinct mechanisms depending on the disease subtype. In STEC-HUS, Shiga toxin binds to Gb3 receptors, which are abundantly expressed on cerebral microvascular endothelial cells [8,9]. This interaction leads to cleavage of 28S rRNA, halting protein synthesis and triggering apoptosis [8,9]. This cascade of events is schematically illustrated in Figure 1 below.

In contrast, in aHUS, endothelial damage is mediated by uncontrolled activation of the complement cascade, particularly the formation of C5b-9, which incorporate into endothelial cell membranes and activate proinflammatory signaling pathways. This disrupts the integrity of the blood–brain barrier [5,16]. In both forms, endothelial dysfunction results in the loss of antithrombotic surface properties and upregulation of adhesion molecules, promoting leukocyte recruitment and local inflammation [11,12].

#### 2.3.2. Microvascular Thrombosis

A prothrombotic state, a hallmark of HUS, emerges directly from endothelial injury. Damaged endothelial surfaces expose subendothelial collagen and release ultra-large von Willebrand factor (vWF) multimers, promoting platelet adhesion and aggregation [12]. Simultaneously, expression of tissue factor is increased, while natural anticoagulants such as thrombomodulin are downregulated, creating optimal conditions for the formation of platelet–fibrin thrombi [11].

When these thrombi occlude small cerebral vessels, the resulting ischemia can lead to cytotoxic and vasogenic edema, ischemic infarctions, and intracranial hemorrhage [21,22,23,24,25]. Neuroimaging often reveals these changes as hyperintensities in regions such as the basal ganglia, thalami, brainstem, and parieto-occipital lobes, frequently correlating with clinical features including PRES, seizures, and altered consciousness [5,26,27,28,29,30,31,32,33,34,35].

#### 2.3.3. Metabolic Encephalopathy

Kidney failure in HUS leads to significant metabolic derangements that secondarily impair CNS function. Uremic toxins, including guanidinosuccinic acid, accumulate and disrupt neuronal activity by interfering with neurotransmitter balance and neuronal excitability [36,37]. Electrolyte imbalances, most notably hyponatremia due to impaired free water excretion, are common precipitants of seizures and altered mental status [38].

Moreover, the systemic inflammatory response associated with HUS results in the release of proinflammatory cytokines such as IL-6 and TNF-α. These cytokines may cross a compromised blood–brain barrier and exert direct neurotoxic effects [12,25,29,39,40]. These metabolic factors often amplify the neurological injury initiated by endothelial damage and thrombosis, providing a plausible explanation for the variable neurological presentations seen in patients with comparable degrees of kidney damage [4].

Although both STEC-HUS and aHUS lead to CNS injury through endothelial dysfunction and microvascular thrombosis, the underlying mechanisms differ significantly. In STEC-HUS, neurological damage is primarily driven by the systemic effects of Shiga toxin, which binds to Gb3 receptors on cerebral endothelial cells, leading to apoptosis and microvascular injury [8,9]. This is often compounded by inflammatory cytokine release and metabolic disturbances due to renal failure. In contrast, aHUS-related CNS injury is largely mediated by uncontrolled activation of the complement system, particularly the formation of the C5b-9, which directly damages endothelial cells and disrupts the blood–brain barrier [5,16].

## 3. Clinical Neurological Manifestations

Neurological complications in HUS encompass a wide range of clinical presentations, with varying onset patterns and severity. They typically emerge during the acute phase of illness. In STEC-HUS, symptoms usually appear between 3 and 7 days after diarrhea onset, with a median of 5 days [5,22,28]. In aHUS, they may occur earlier, often in parallel with renal dysfunction [17]. These manifestations vary in severity and type, ranging from diffuse encephalopathy to focal deficits and movement disorders.

### 3.1. Encephalopathy

Encephalopathy is among the most common neurological manifestations of HUS, particularly in severe clinical cases [4]. It is characterized by an altered or fluctuating level of consciousness [5], with a clinical spectrum ranging from mild confusion and irritability to profound lethargy, stupor, or coma. Neurological symptoms frequently emerge several days after the onset of diarrheal illness, which may complicate timely recognition of the underlying etiology. Although encephalopathy is not universally present, its occurrence necessitates prompt diagnosis and intervention due to the potential for severe systemic complications requiring intensive care or resulting in fatal outcomes [39].

### 3.2. Seizures and Status Epilepticus

Seizures are a significant neurological complication of HUS, occurring in approximately 25% of patients who present with central nervous system (CNS) involvement [40]. Although rare, progression to chronic epilepsy has been documented, with only two studies to date reporting such long-term sequelae [29,36].

Status epilepticus (SE), a more severe manifestation, has been described in multiple pediatric case reports [5,30,31,32,33]. In many of these cases, SE was either the first or an early indicator of CNS involvement. In more severe or atypical presentations, electroencephalographic (EEG) abnormalities and findings consistent with PRES have also been observed. Clinical outcomes vary widely, ranging from full neurological recovery to persistent deficits and long-term sequelae [5,30,31,32,33].

### 3.3. Focal Neurologic Deficits

Focal neurological deficits in HUS are relatively uncommon but can occur across all age groups. Key clinical indicators include hemiparesis, aphasia and stroke-like syndromes [5,38,41,42,43,44,45,46,47].

Through systematic review, multiple case reports have documented focal deficits in both pediatric and adult populations. In children, these events have been observed in association with both typical (STEC-related) and atypical HUS. They are rarely isolated findings and are often accompanied by more common features such as seizures and encephalopathy. Reported manifestations include hemiparesis, cerebral infarction with ataxia, expressive aphasia, ophthalmoplegia, multifocal cerebral ischemia and internal carotid artery (ICA) stenosis [5,38,41,42,43,44].

In adult patients, focal deficits are more frequently linked to atypical HUS, typically driven by complement dysregulation or the presence of anti-complement antibodies [45,46]. Clinical presentations in this group often mirror those seen in pediatric cases and include hemiparesis, multifocal ischemic strokes and episodes of confusion [45,46,47].

### 3.4. Movement Disorders

Movement disorders associated with HUS are most frequently reported in pediatric patients, particularly in cases of atypical HUS [29,48]. These manifestations are believed to result from the accumulation of uremic toxins due to kidney damage, which can contribute to metabolic encephalopathy. Neuroimaging often reveals bilateral involvement of the basal ganglia, a characteristic finding in these cases. These disturbances are typically transient and resolve as the underlying condition improves [29,48].

### 3.5. Cortical Blindness and PRES

PRES may develop several days after the onset of HUS and has been documented in both typical and atypical subtypes. MRI findings typically reveal vasogenic edema predominantly in the parieto-occipital lobes [2,8,34,35], although more extensive signal changes involving the basal ganglia, thalami, and brainstem have also been reported.

Clinical manifestations of PRES are diverse and include seizures, altered mental status, headache, and various visual disturbances, ranging from cortical blindness to visual field deficits and hallucinations [2,8,34,35]. Importantly, the visual loss seen in PRES originates from occipital lobe dysfunction, rather than from ocular pathology.

PRES in the context of HUS is often associated with precipitating factors such as hypertension, eclampsia or preeclampsia, immunosuppressive therapy, chronic kidney disease (CKD), and autoimmune conditions [8].

## 4. Diagnostic Workup

Comprehensive diagnosis and timely suspicion of neurologic involvement in HUS is essential, given the likelihood of rapid deterioration and diverse clinical presentation.

### 4.1. Laboratory Evaluation

Due to the variable clinical presentation of HUS, laboratory assessment must be broad and thorough, encompassing hematologic, renal, metabolic, inflammatory and microbiological parameters [4,5,21,29,39,40].

Key findings on complete blood count (CBC) include MAHA and thrombocytopenia, both of which are essential in the context of neurologic assessment. Anemia may exacerbate neurological symptoms through tissue hypoxia [5]. Hemolytic anemia, commonly observed in HUS, is reflected by elevated lactate dehydrogenase (LDH) and reduced haptoglobin levels, ADAMST13, and is closely linked to endothelial injury—a potential contributor to neurologic manifestations [39].

Assessment of kidney function, including blood urea nitrogen (BUN) and serum creatinine, is necessary to determine the degree of kidney impairment [4,5]. These markers are also relevant for evaluating the risk of metabolic encephalopathy, which may worsen neurological status [40].

Electrolyte disturbances should be promptly identified. Hyponatremia, often resulting from impaired free water clearance, is a known precipitant of seizures and altered mental status [38]. Hyperkalemia and hypocalcemia can also contribute to neurologic dysfunction [49].

Systemic inflammatory markers such as erythrocyte sedimentation rate (ESR) and C-reactive protein (C-RP) are frequently obtained in routine practice. However, more specific indicators of CNS inflammation, such as IL-6 and TNF-α, though relevant, are not routinely measured [12,29,38].

Evaluation of acid–base balance is also important, as metabolic acidosis is a common finding in HUS and may contribute to neuronal excitability and neurological symptoms [50]. 

Critically, microbiological testing is required to confirm typical STEC-HUS, with a stool culture serving as the gold standard for confirming Shiga toxin-producing *E. coli* [51]. Presently, real-time Polymerase Chain reaction (PCR) analyses of Stx genes with high efficiency and accuracy are available. Because real-time PCR can quickly yield a result, it can be used to first detect Stx while waiting for stool cultures to confirm the diagnosis [52]. Real-time PCR is also valuable for distinguishing between typical and atypical cases, which may differ in clinical course and neurologic involvement [51]. Therefore, microbiological testing for STEC-HUS ideally includes a combination PCR and stool culture for a high detection rate [52].

### 4.2. Neuroimaging

Neuroimaging has an important role in the evaluation of neurologic involvement in HUS. The preferred modality is magnetic resonance imaging (MRI), due to its superior sensitivity than computer tomography (CT). A frequently reported MRI finding is bilateral involvement of the basal ganglia; however, the imaging spectrum is broad and reflects the heterogeneity of clinical manifestations. For instance, in cases of PRES, MRI typically reveals vasogenic edema predominantly affecting the parieto-occipital lobes, although additional involvement of the basal ganglia, thalami, and brainstem may also be observed [34,35].

CT remains valuable in the acute setting, particularly for the rapid exclusion of intracranial hemorrhage or large territorial infarctions [21]. Recent advances in MRI techniques, such as diffusion-weighted imaging (DWI) and susceptibility-weighted imaging (SWI), have further enhanced diagnostic capabilities by enabling detection of microhemorrhages and cytotoxic edema, which may not be visible on conventional sequences [53,54].

In addition to the initial imaging assessment, serial MRI follow-up is recommended to monitor lesion evolution and treatment response. Among various MRI findings, the presence of a hemorrhagic component within an acute lesion is the most consistent predictor of residual abnormalities on follow-up imaging [21].

### 4.3. EEG and Neurophysiology

Electroencephalography (EEG) is a valuable diagnostic tool in the evaluation of neurological involvement in HUS, particularly in patients presenting with seizures, altered mental status, or encephalopathy [55,56]. The most frequently observed EEG abnormalities include focal epileptiform discharges, diffuse slowing, and periodic patterns. Although these findings are often nonspecific, they frequently correlate with clinical and radiologic data, aiding in diagnosis and disease monitoring [29,48].

In patients with encephalopathy or at risk for subclinical seizures, continuous EEG monitoring is recommended for early detection and management of electrical disturbances [57].

Following the acute phase, patients recovering from HUS with neurological complications may benefit from neurophysiological assessment. Such evaluations can identify persistent cognitive impairments, particularly in domains such as memory, attention, and executive function [58]. 

**Figure 2:**
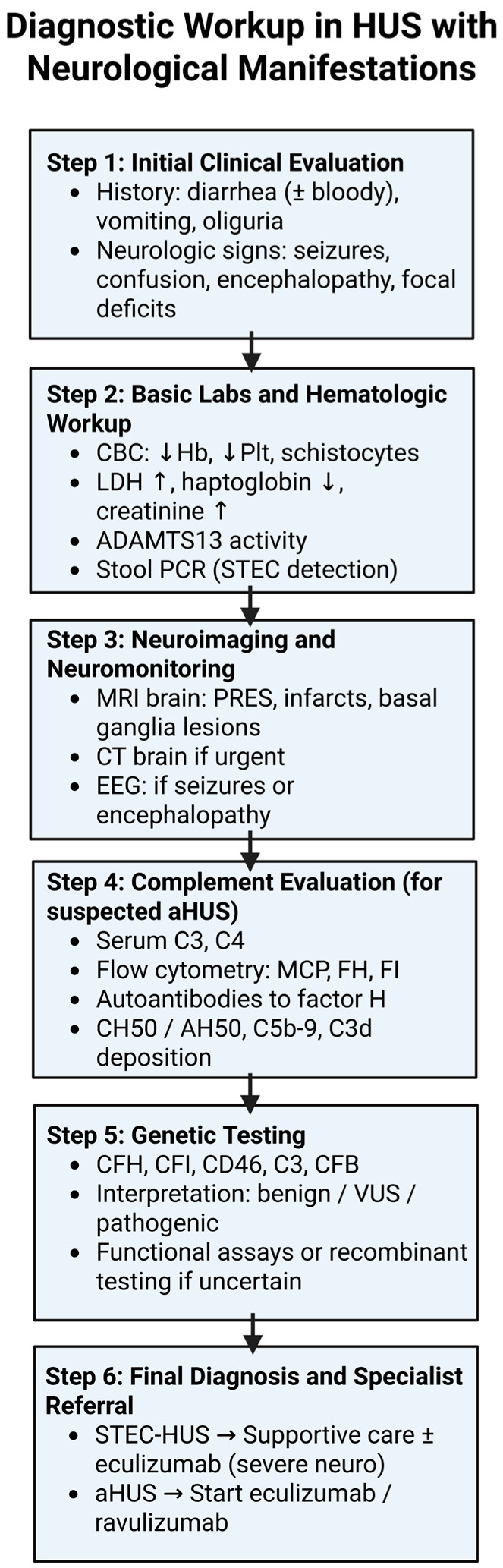
Diagnostic workup in HUS with neurological manifestations. A stepwise algorithm outlining the evaluation of HUS patients with neurological symptoms, including clinical assessment, laboratory testing, neuroimaging, complement studies, genetic analysis, and final diagnostic differentiation between STEC-HUS and aHUS. Created in BioRender online application, available at https://app.biorender.com/ (accessed on 10 June 2025).

The stepwise diagnostic approach to evaluating neurological involvement in patients with HUS is presented in Figure 2 below.

## 5. Therapeutic Approaches

The treatment of HUS is complex and combines various therapeutic modalities. The primary goal is to stabilize the patient, prevent further damage, and manage complications. Therapy is tailored based on the severity of symptoms and the underlying cause of HUS, with a focus on both acute and long-term management.

### 5.1. Supportive Care

Supportive care in HUS comprises several critical interventions aimed at maintaining patient stability during the acute phase. This includes meticulous fluid and electrolyte management to prevent dehydration and protect kidney function [59]. It also involves measures to prevent micro- and macrovascular thrombotic events. As a result, prophylactic low-molecular-weight heparins (LMWH) are frequently administered to reduce the risk of cerebral infarction and associated complications [60].

Effective blood pressure control is essential, often achieved with agents such as renin-angiotensin system blockers, alpha and beta blockers, or calcium channel antagonists, depending on the clinical presentation. However, in the presence of acute kidney injury, potassium-sparing diuretics and alpha or beta blockers should be avoided due to their potential to exacerbate hyperkalemia and compromise kidney perfusion [61].

Nutritional support, including total parenteral nutrition, becomes vital when gastrointestinal function is impaired, ensuring adequate caloric intake and preventing catabolic complications. Continuous monitoring of organ function, particularly kidney, is crucial to detect any worsening of kidney parameters. In cases of AKI with rising nitrogenous waste products, prompt initiation of hemodialysis (HD) is required to manage electrolyte imbalances and reduce [62].

In selected severe cases, additional supportive measures such as intravenous immunoglobulin (IVIG) and continuous hemodiafiltration (CHDF) may be considered. IVIG has been used in a case of aHUS with complement mutation, successfully managed with steroids and IVIG without plasma exchange. CHDF has been employed post-transplant in unstable patients to continuously clear uremic toxins and inflammatory mediators [63,64].

### 5.2. Antimicrobial Agents

In cases of suspected HUS, especially when STEC infection is a potential underlying cause, empiric antibiotic therapy is generally not recommended prior to establishing the diagnosis. Early use of certain antibiotics, particularly trimethoprim-sulfamethoxazole, metronidazole, and some β-lactams, has been linked to an increased risk of developing HUS in patients with STEC infection. This is thought to occur due to enhanced release of Shiga toxin during bacterial lysis, potentially exacerbating endothelial damage. Therefore, antibiotics such as tetracyclines and fluoroquinolones, which may pose additional safety risks or adverse effects, are typically avoided [65]. However, if a patient’s clinical status necessitates empiric antibiotic coverage, agents with a lower association with HUS risk, such as penicillins, cephalosporins, or macrolides like azithromycin, may be cautiously considered, although their use remains controversial in the context of STEC infections. Antibiotic selection should also be informed by patient-specific factors, such as age, comorbidities, and pregnancy status, while parallel diagnostic efforts aim to confirm the etiology and guide targeted therapy [66].

### 5.3. Plasma Exchange

Plasma exchange (PEX) is a procedure used to remove harmful substances from the blood by exchanging the patient’s plasma with donor plasma or a plasma substitute. It is commonly used in conditions such as aHUS, particularly when caused by anti-factor H (FH) antibodies, or when access to complement inhibitors like eculizumab is limited. PEX aims to rapidly reduce circulating pathogenic factors and has shown reasonable short-term effectiveness, especially in anti-FH aHUS. Its duration is typically empirical, guided by clinical response and laboratory markers such as platelet count, LDH, and hemoglobin levels. A recent study by Thangaraju et al. demonstrated that an abbreviated PEX protocol was equally effective as standard treatment, with comparable short-term outcomes and reduced anti-FH titers [67]. Another study by Khandelwal et al. reported a hematologic remission rate of 96.3% after a median of six PEX sessions and found that the majority of patients achieved dialysis independence within 3 months [68].

### 5.4. Eculizumab and Complement Inhibitors

Eculizumab is a monoclonal antibody that blocks complement component C5, preventing the formation of the MAC. This inhibition protects blood vessel cells from damage caused by uncontrolled complement activation, which is a key factor in aHUS. Eculizumab is used mainly to treat aHUS patients with genetic or acquired complement system abnormalities. It starts working within days to weeks, improving blood and kidney function. Maintenance doses are given every 2 to 4 weeks to sustain its effect. Common side effects include headache, high blood pressure, and increased risk of infections, especially meningococcal infections, so vaccination is required before treatment. Neurological symptoms in HUS may improve with eculizumab, but results vary. Newer drugs like ravulizumab work similarly but need less frequent dosing. Wildes et al. (2024) presented a case series of four pediatric patients with STEC-HUS and CNS involvement who were treated exclusively with eculizumab [69]. All patients experienced resolution of neurological symptoms within 24 to 72 h after administration and achieved complete kidney and neurological recovery at 12-month follow-up [69].

In a multicenter retrospective study, Percheron et al. (2018) analyzed 33 children with severe STEC-HUS treated with eculizumab, of whom 28 had neurological symptoms [70]. Among these, 19 patients showed favorable neurological outcomes, including 17 with prompt recovery following the first eculizumab injection [70].

Additionally, a systematic review and meta-analysis by Mahat et al. evaluated the role of eculizumab in improving neurological outcomes in pediatric patients with severe STEC-HUS. The review found that while eculizumab may offer benefits for specific subgroups, current evidence does not support its routine use to improve neurological outcomes, highlighting the need for high-quality randomized controlled trials [71].

Ravulizumab (Ultomiris) is a long-acting monoclonal antibody approved by the FDA in 2019 for the treatment of aHUS in both adult and pediatric patients aged one month and older. It functions by inhibiting terminal complement component C5, thereby preventing complement-mediated TMA [72,73].

It is also important to point out that all patients receiving complement-targeted therapeutics like the above-mentioned eculizumab and ravulizumab are at a higher risk of infections with encapsulated bacteria and must therefore receive a meningococcal vaccine prior to treatment [74].

The high cost of eculizumab and ravulizumab remains a substantial barrier to broader clinical use, particularly in resource-limited settings. Annual treatment expenses may reach over USD 500,000 per patient, emphasizing the need for careful patient selection and individualized therapy plans to ensure cost-effectiveness and sustainable access [75].

In patients with aHUS who progress to end-stage renal disease (ESRD), kidney transplantation may be considered. However, the risk of post-transplant recurrence varies significantly depending on the underlying genetic abnormality. Mutations in *CFH*, *CFB* and *C3* are associated with high recurrence rates and poor graft outcomes, while *MCP (CD46)* mutations are linked to lower recurrence risk and more favorable prognosis [14,15]. Pre-transplant genetic screening is essential for risk stratification and guiding therapy decisions, including consideration of complement blockade in high-risk patients.

### 5.5. Neurologic Symptom Management

Anticonvulsants are commonly employed as first-line treatment due to their rapid effectiveness in controlling seizures, with agents like benzodiazepines often used initially. Additional antiepileptic medications such as levetiracetam or phenytoin may be necessary depending on the patient’s clinical response and the severity of the neurological manifestations. When selecting and dosing these medications, special consideration must be given to the patient’s renal function, as reduced glomerular filtration, especially in the context of dialysis, significantly affects drug clearance. For example, dosing should be adjusted as if the glomerular filtration rate is approximately 10 mL/min/1.73 m^2^ to avoid drug accumulation and toxicity [76].

Supportive neurological management in HUS includes not only symptom control but also prevention of complications, such as microangiopathic and macroangiopathic thromboses. For this reason, prophylactic administration of LMWH is often considered, particularly to reduce the risk of cerebral infarction and related neurological symptoms.

### 5.6. Neurorehabilitation and Follow-Up

Neurorehabilitation and long-term follow-up are essential components in the management of HUS with neurological involvement. Early recognition of neurological complications, such as seizures or encephalopathy, allows timely intervention and improves outcomes. A multidisciplinary neurorehabilitation approach typically includes physical, occupational, speech, and cognitive therapies aimed at addressing motor, functional, communication, and cognitive deficits. Medications may also be necessary to control symptoms like seizures or muscle spasms. Long-term follow-up involves regular neurological assessments, kidney function monitoring, and blood pressure control to detect and manage chronic complications [77].

Psychological support is often important to help patients cope with emotional and cognitive challenges. The severity of neurological sequelae varies, with some patients achieving full recovery while others require prolonged rehabilitation and monitoring.

## 6. Prognosis and Outcomes

### 6.1. Short-Term Neurological Outcomes

In the acute phase of HUS, neurological complications often include seizures and status epilepticus, the latter reflecting widespread cortical irritation or infarction. Altered mental status may also be accompanied by focal neurological signs such as hemiparesis, cranial nerve palsies, or aphasia. Visual disturbances including diplopia or cortical blindness are typically associated with occipital lobe edema or involvement of the posterior circulation. Additional manifestations may include movement disorders, psychiatric symptoms such as hallucinations or agitation, and signs of both pyramidal and extrapyramidal involvement. MRI and neuropathological studies have confirmed that CNS injury in HUS can be extensive, often revealing ischemia, edema, and hemorrhage, particularly in the cerebral cortex and thalamus. Hypertension (HTN) secondary to CKD may exacerbate encephalopathy and contribute to the development of PRES [33,34,35,36,40,78].

Overall, CNS involvement signals a more severe short-term clinical course and often necessitates intensive care management.

### 6.2. Long-Term Neurological Outcomes

Long-term sequelae may persist in patients who experienced severe neurological complications during the acute phase. These include chronic epilepsy, hemiparesis, visual impairment and cognitive deficits. Although global intellectual disability is now rare, many patients experience subtler impairments such as reduced fine motor coordination, balance disturbances, or impaired motor sequencing. In one study, children who had suffered CNS involvement during HUS retained normal intelligence quotients (IQs) but displayed mild neuromotor deficits that affected handwriting and balance, findings that often go unnoticed without formal neurodevelopmental testing [5,77].

Behavioral and attentional problems are also reported. Qamar et al. described pediatric survivors who demonstrated symptoms of inattention, hyperactivity, and clumsiness despite performing within normal limits on standard cognitive assessments. These findings underscore the potential for lingering neurodevelopmental effects, even in children who appear to recover fully [79].

Long-term monitoring is particularly crucial for infants and young children. Those who experienced coma or stroke during the acute illness are at elevated risk for developmental delays and learning difficulties. Even asymptomatic children may benefit from neuropsychological assessment, as routine clinical evaluations may fail to detect subtle impairments. Tools such as the Pediatric Cerebral Performance Category are useful in general follow-up but may not adequately capture behavioral or cognitive changes, reinforcing the need for more sensitive diagnostic approaches [33,80].

Adult survivors of severe HUS frequently report persistent cognitive complaints, depression and anxiety. In pediatric populations, behavioral problems can negatively impact academic performance and social development. Patients with aHUS, in particular, face a chronic disease course with potential relapses, which can add significantly to psychological distress [81,82].

The emotional burden also extends to caregivers and families, who may experience significant psychological strain during and after the illness. For this reason, comprehensive follow-up care should include not only neurological rehabilitation, but also psychological and social support. Counseling, behavioral therapy, and educational interventions may all play a critical role in optimizing long-term outcomes [81,82,83].

## 7. Age-Related Differences in Neurological Involvement of HUS

### 7.1. Prevalence, Clinical Patterns and Imaging Differences

Neurological complications in STEC-HUS occur in both children and adults, though prevalence and presentation vary. In pediatric cohorts, studies reported acute neurological complications in approximately 10–11% of cases, most commonly seizures and encephalopathy [5,84]. In contrast, during specific adult outbreaks such as the 2011 German STEC O104:H4 epidemic, neurological complications were reported in up to 100% of cases, often with focal symptoms such as diplopia, word-finding difficulties, and accentuated reflexes [56,58].

Long-term sequelae also differ. While only 4% of all pediatric STEC-HUS patients develop lasting neurological deficits, the rate rises to 50% among those with initial CNS involvement, manifesting as epilepsy, cortical blindness, or hemiparesis [85,86,87]. Adults, though sometimes reported to recover completely [56,58], may show persistent deficits on neuropsychiatric testing, including psychomotor slowing and impaired concentration [58,88].

Neuroimaging studies reveal age-specific differences. Pediatric cases often show bilateral symmetric basal ganglia lesions and cortical-subcortical involvement [29,89,90], while adults more frequently exhibit brainstem vasogenic edema or PRES-like changes [91,92]. Hemorrhage is more common in pediatric HUS, while rare in adults [89,90].

In aHUS, CNS involvement is a predominant extra-renal manifestation in both age groups. Pediatric registry data indicate a 27.2% prevalence, mainly with seizures [93], while adult studies report lower rates (~35%), while adult studies report neurological symptoms in up to 35% of aHUS cases, including motor symptoms and visual changes [92].

### 7.2. Therapeutic Implications and Outcomes by Age

While supportive therapy remains the cornerstone of management in both age groups, treatment strategies and outcomes may differ. In pediatric STEC-HUS with neurological involvement, interventions such as plasma exchange and eculizumab have been associated with favorable recovery. In one cohort, 91% of children receiving these therapies achieved complete neurological resolution [5]. However, a French multicenter study found no significant difference in one-year neurological outcomes across treatment modalities, suggesting that advanced therapies may be reserved for severe or refractory cases [94].

Adult patients with STEC-HUS, particularly during outbreak scenarios, have shown mixed responses to acute-phase therapies. Although initial neurological improvement is often observed, long-term follow-up reveals lingering cognitive symptoms in many cases, emphasizing that full recovery is not guaranteed [95,96].

For aHUS, eculizumab is the recommended first-line treatment across all ages, with well-documented efficacy in improving kidney and neurological outcomes and reducing TMA events. Long-term studies suggest potential benefit in minimizing neuropsychiatric sequelae, but further large-scale follow-up is warranted [97,98,99,100].

## 8. Conclusions

Neurologic complications in HUS, though less frequent today, remain a major contributor to morbidity and mortality, particularly in pediatric populations. Diverse clinical presentations and evolving imaging patterns require prompt recognition and diagnostic approaches. Despite improved outcomes with supportive care and complement inhibition in aHUS, long-term neurocognitive and psychosocial consequences may persist, often undetected without formal testing. Future progress depends on earlier risk identification, wider access to targeted therapies, and standardized long-term follow-up strategies to prevent irreversible CNS damage and optimize recovery.
